# Expression and Functional Studies of INS-5, an Insulinase-Like Protein in *Cryptosporidium parvum*

**DOI:** 10.3389/fmicb.2020.00719

**Published:** 2020-05-08

**Authors:** Ni Ni, Ruilian Jia, Yaqiong Guo, Na Li, Haizhen Wu, Yaoyu Feng, Lihua Xiao

**Affiliations:** ^1^State Key Laboratory of Bioreactor Engineering, School of Resource and Environmental Engineering, East China University of Science and Technology, Shanghai, China; ^2^Key Laboratory of Zoonosis of Ministry of Agriculture, College of Veterinary Medicine, South China Agricultural University, Guangzhou, China; ^3^School of Biotechnology, East China University of Science and Technology, Shanghai, China

**Keywords:** *Cryptosporidium parvum*, insulinase, recombinant protein, invasion, expression

## Abstract

The small *Cryptosporidium* genome (∼9 Mb) has over 20 copies of genes encoding insulinase-like proteases (INS), suggesting that these enzymes may have important biological functions in the pathogen and could be developmentally regulated. In this study, INS-5, a unique member of the INS family in *Cryptosporidium parvum*, was cloned and expressed in *Escherichia coli* BL21 (DE3). In addition to the predicted INS-5 of ∼78 kDa, smaller fragments of ∼70, ∼55, and ∼30 kDa were simultaneously generated. After purification through a nickel-nitrilotriacetic acid affinity column, the full recombinant protein obtained was used to prepare polyclonal antibodies. Antibodies raised against INS-5 recognized the recombinant protein and native protein in sporozoite extracts. Further characterization of INS-5 included qRT-PCR assessment of gene expression; immunofluorescence localization of the protein expression in sporozoites, merozoites, and other developmental stages; and neutralization of invasion of *C. parvum in vitro*. The results obtained indicated that although INS-5 was expressed in sporozoites and merozoites, the high gene expression was from 36 to 48 h of the *in vitro* culture after invasion. Anti-INS-5 antibodies partially neutralized the invasion (inhibition rate = 38.5%). Results of this study suggest that INS-5 plays some role in the invasion and growth of *C. parvum*.

## Introduction

*Cryptosporidium* spp. are apicomplexan parasites of the gastrointestinal epithelium, causing diarrhea in humans and various animals (Checkley et al., 2015). Over 40 *Cryptosporidium* species have been described (Feng et al., 2018). Among them, *Cryptosporidium parvum* and *Cryptosporidium hominis* are the most common species reported in humans (Xiao, 2010). Most work to date on the biology and pathogenicity of *Cryptosporidium* spp., however, was done using *C. parvum* (Bhalchandra et al., 2018).

The invasion process of *Cryptosporidium* spp. is not fully understood (Yoshida et al., 2011; Singh et al., 2015). The first step of the invasion, oocyst excystation, is triggered by changes in the external environment such as temperature and pH, with the infective sporozoites being released following enzymolysis of the oocyst wall (Borowski et al., 2008). The apical organelles of sporozoites discharge a variety of molecules that are required in host–cell attachment and invasion (Lendner and Daugschies, 2014). Upon attachment to the host cell, sporozoites initiate host–cell membrane protrusion, forming a parasitophorous vacuole (PV) that encapsulates the parasite. The *Cryptosporidium* molecules possibly involved in host cell attachment and invasion include GP40/15, GP900, P23, TRAP, and CSL (Singh et al., 2015). How these proteins are processed during sporozoite invasion, however, remains poorly elucidated. Whole-genome sequencing of *C. parvum* has identified over 20 insulinase-like proteases (INS) (Abrahamsen et al., 2004; Guo et al., 2015). In particular, 12 INS genes are present in tandem in the 3′ subtelomeric region of chromosome 3, suggesting the likelihood of regulated expression of INS proteins (Mauzy et al., 2012). Most of these INS are present in other intestinal *Cryptosporidium* species. As only 2% of *Cryptosporidium* genes have multiple copies, the expansion of this gene family suggests that the INS may have important biological functions during the invasion and development of *Cryptosporidium* spp. (Liu et al., 2016).

Insulinase-like protease proteases are currently classified as belonging to the M16A zinc metalloproteinase subfamily defined by an “inverted” HXXEH active site motif (Fernández-Gamba et al., 2009). Studies have shown that INS proteases have broad substrate specificity and are localized in the cytosol, peroxisomes, endosomes, and even on the surface of cells, perhaps as a reflection of the diverse biological functions of these enzymes (Laliberté and Carruthers, 2011). For example, *Vibrio vulnificus* secretes a novel insulinase, SidC, which contributes to the proliferation of this human bacterial pathogen (Kim et al., 2015). An M16A enzyme in yeasts, Ste23p, proteolyzes mammalian substrates Aβ1–40 and insulin B-chain (Alper et al., 2010). In apicomplexan parasites, falcilysin and toxolysins, INS of *Plasmodium falciparum* and *Toxoplasma gondii*, respectively, are important in the development and fitness of parasites (Ponpuak et al., 2007; Laliberté and Carruthers, 2011). The biological functions of INS in *Cryptosporidium* spp. are not clear but are expected to be diverse because of the high number of INS genes with diverse sequences (Mauzy et al., 2012).

In this study, we conducted some preliminary characterization of the INS-5 protein encoded by the *cgd2_2760* gene in *C. parvum*. This INS differs from the other INS in *Cryptosporidium* spp. by having only one inactive domain of the four domains associated with active INS proteases.

## Materials and Methods

### Oocysts, Cells, Plasmids, *Escherichia coli* Strains, and Culture Conditions

*Cryptosporidium parvum* oocysts (IOWA isolate) were purchased from Waterborne, Inc. (New Orleans, LA, United States) and stored in antibiotics at 4°C for less than 2 months before use. They were treated with 0.5% sodium hypochlorite for 10 min on ice and exposed to excystation solution containing 0.75% taurodeoxycholic acid and 0.25% trypsin at 37°C for 1 h to obtain free sporozoites. Human ileocecal adenocarcinoma HCT-8 cells (ATCC CCL-244) were obtained from the Shanghai Branch of the Chinese Academy of Sciences and cultured in RPMI 1640 medium supplemented with 10% fetal bovine serum (FBS) and 1% penicillin–streptomycin solution (PS) at 37°C under 5% CO_2_. The pET28a vector was obtained from Novagen, Inc. (Madison, WI, United States), while *Escherichia coli* strains DH5α and BL21 (DE3) were obtained from Tiangen Biotech (Beijing, China).

### Construction of Recombinant Plasmid

Polymerase chain reaction (PCR) was used to amplify the *cgd2_2760* gene from the genomic DNA of the *C. parvum* IOWA isolate. The primers used included 5′-CGGGATCCATGACAGCTAATGGGAA-3′ (the added restriction site *Bam*HI is underlined) and 5′-ACGCGTCGACAAGACCTAAGTCGC-3′ (the added restriction site *Sal*I is underlined) and were synthesized by Sangon Biotech, Co., Ltd. (Shanghai, China). The template DNA was extracted from *C. parvum* oocysts by using the QIAGEN DNeasy Blood & Tissue Kit (Qiagen, Hilden, Germany). The PCR cycle included one cycle of 95°C for 5 min; 35 cycles of 95°C for 45 s, 55°C for 45 s, and 68°C for 2 min; and one cycle of 68°C for 7 min. The PCR was performed in a GeneAmp 9700 (Applied Biosystems, Foster City, CA, United States). The amplified PCR product was purified using the SanPre PCR Product Purification Kit (Sangon Biotech) and digested with restriction enzymes *Bam*HI and *Sal*I. The resulting fragment was inserted between the *Bam*HI and *Sal*I restriction sites of pET28a vector to generate recombinant plasmid *cgd2_2760*-pET28a, which was transformed into the competent *E. coli* DH5α. Positive colonies selected on LB agar with kanamycin were identified by PCR and sequenced to confirm their identity and sequence accuracy.

### Expression and Purification of INS-5

The *cgd2_2760*-pET28a plasmid was isolated from *E. coli DH5*α using the EZ-10 Spin Column Plasmid DNA Minipreps Kit (Sangon Biotech). *E. coli* BL21 (DE3) cells were transformed with the plasmid and grown in LB medium supplemented with 100 μg/ml of kanamycin at 37°C. When the *A*_600_ of the culture reached 0.6–0.8, the expression of the recombinant INS-5 protein was induced by adding 0.5 mM isopropyl-β-D-thiogalactoside (IPTG). The *E. coli* cells were cultured at 16, 25, and 37°C for another 5 h to identify the most favorable temperature for the expression of the recombinant protein. This led to the section of 25°C as the optimal temperature.

For the production of recombinant protein, *E. coli* BL21 cells were inoculated into 1-L medium and cultured as described above. The cells were harvested by centrifugation at 4°C and 8,000 × *g* for 20 min, resuspended in phosphate-buffered saline (PBS) at pH 7.4, and lysed by sonication on ice. The insoluble components were collected by centrifugation at 4°C and 10,000 × *g* for 20 min. The pellet containing inclusion bodies was dissolved in 8M urea. After centrifugation at 4°C and 10,000 × *g* for 20 min, the supernatant containing the denatured recombinant protein was filtered through a 0.45-μm cellulose acetate membrane filter (Millipore, Norcross, GA, United States) and applied to a 1-ml nickel-nitrilotriacetic acid (Ni-NTA) affinity column (Novagen). The column was washed with five volumes of equilibration buffer and eluted with six volumes of 500 mM imidazole in 8M urea. These steps were repeated six times to obtain enough target protein of high purity. The renaturation of the purified protein was achieved by gradient dialysis from urea solution to PBS buffer at 4°C. The renatured protein was concentrated by ultrafiltration with Amicon^®^ Ultra-15 10K Centrifugal Filter Devices (Millipore) and assessed for purity using SDS-PAGE stained with Coomassie Brilliant Blue and western blot analyses. The bands of the expected size and lower-molecular-weight species were excised from the gel and analyzed using matrix-assisted laser desorption/ionization time of flight mass spectrometry (MALDI-TOF-MS).

### Preparation of Polyclonal Antibodies

Polyclonal antisera were raised by GenScript, Co., Ltd. (Nanjing, China) using immunization of rabbits with the purified INS-5 protein of ∼78 kDa dissolved in PBS buffer at the concentration of 0.35 mg/ml. The sera were collected from the immunized rabbits and used in the purification of polyclonal IgG antibodies through an affinity chromatographic column conjugated with recombinant INS-5. The final concentration of polyclonal antibodies was 0.4 mg/ml. The specificity of the antibodies was evaluated using western blot analysis.

### Western Blot Analysis

Western blot analysis was used to assess the purity of the recombinant INS-5 protein. The INS-5 protein was electrophoresed on 10% SDS polyacrylamide gels and transferred onto a polyvinylidene fluoride (PVDF) membrane. After blocking with 5% non-fat milk in PBS and 0.5% Tween 20 (PBST) for 2 h, the membrane was probed with anti-His tag antibodies (Cell Signaling Technology, Danvers, MA, United States) at 1:1,000 dilution for 2 h. After incubation with horseradish peroxidase (HRP)-conjugated goat anti-mouse antibodies (Yeasen, Shanghai, China) at 1:5,000 dilution for 1 h, the antigen–antibody complex was visualized using the DAB kit (Tiangen Biotech). The membrane was washed three times with PBST after each incubation.

The native INS-5 extracted from *C. parvum* oocysts was also assessed by western blot analysis (∼5 × 10^6^ oocysts/lane), using the procedure described above. Anti-INS-5 polyclonal antibodies (0.16 μg/ml), immune sera (1:2,500) or pre-immune sera (1:2,500), were the primary antibodies, and HRP-conjugated goat anti-rabbit antibodies (Yeasen) (1:5,000) were the secondary antibodies in western blot analysis. Crude native proteins were released from free sporozoites after boiling them in 5 × protein loading buffer for 5 min.

### Localization of INS-5 Expression in Developmental Stages by Immunofluorescent Microscopy

HCT-8 cells were seeded into a plastic 12-well plate with coverslips, grown to 40–50% confluence, and exposed to *C. parvum* oocysts that had been treated with 0.5% sodium hypochlorite as described above. The cultures were maintained at 37°C for 24 or 48 h to examine intracellular stages and for 36 h to collect free merozoites from the culture. The infected cultures, merozoites, oocysts, and free sporozoites were fixed at room temperature with methanol for 15 min and permeabilized with 0.5% Triton X-100 in PBS for 15 min. After blocking of non-specific binding with 5% BSA in PBS at room temperature for 1 h, the slides were incubated with anti-INS-5 antibodies (0.4 μg/ml) in 5% BSA–PBS for 1 h and Alexa Fluor^®^ 594-conjugated goat anti-rabbit IgG (Cell Signaling Technology) in BSA–PBS at 1:400 dilution for another hour. After counterstaining with the nuclear stain 4′,6-diamidino-2-phenylindole (DAPI, Roche, Basel, Switzerland) at 1:2,000 dilution for 10 min, the slides were mounted with No-Fade Mounting Medium (Booster, Wuhan, China) and examined using differential interference contrast (DIC) and fluorescence microscopy on a BX53 microscope (Olympus, Tokyo, Japan).

### Evaluation of Expression of *cgd2_2760* Gene by qPCR

Quantitative PCR (qPCR) was used to evaluate the expression of the *cgd2_2760* gene in developmental stages of *C. parvum*. Data on the expression of *C. parvum* 18S rRNA (Cp18S rRNA) gene were used in data normalization. In this analysis, HCT-8 cells were seeded into a 12-well plate, grown to 80–90% confluence, and exposed to sodium hypochlorite-treated *C. parvum* oocysts. After the invasion, infected HCT-8 cells were collected at 2, 6, 12, 24, 36, 48, and 72 h of the *in vitro* culture. Total RNA was extracted from wells using an RNeasy Mini Kit (Qiagen) and measured for concentration using a NanoDrop 2000 (Thermo, Waltham, MA, United States). cDNA was synthesized from 1 μg of the extracted RNA using a GoScript^TM^ Reverse Transcription System (Promega, Beijing, China) and analyzed by qPCR in a LightCycler^®^ 480 (Roche), with the following cycling conditions: 95°C for 3 min and 45 cycles of 95°C for 30 s, 58°C for 30 s, and 72°C for 30 s. The 20-μl qPCR reaction contained 1 μl of cDNA, 0.1 mM primers, and 10 μl of SYBR Green PCR Mix (TOYOBO, Osaka, Japan). The primers used included 5′-GAATTTGGAATTTGGGATGC-3′ and 5′-GCTCGCATGAATCATTTTGA-3′ (amplicon size = 220 bp) for the analysis of the *cgd2_2760* gene, and the published qPCR primers 5′-CTC CAC CAA CTA AGA ACG GCC-3′ and 5′-TAG AGA TTG GAG GTT GTT CCT-3′ (amplicon size = 256 bp) from the Cp18S rRNA gene (Awad-El-Kariem et al., 1994). The data generated were from three culture replicates of three independent experiments. The relative expression of the target gene at different time points was calculated by using the 2^–^^△^^△^^*CT*^ method (Livak and Schmittgen, 2001).

### Invasion Neutralization Assay

*Cryptosporidium parvum* oocysts treated with 0.5% sodium hypochlorite were incubated at 37°C for 15 min in medium containing pre-immune serum or immune serum diluted at 1:100, 1:200, 1:500, and 1:1,000, with medium alone as controls. Oocysts and excysted sporozoites in 100 μl were seeded onto HCT-8 cells cultured to 80–90% confluence in a 12-well plate (with 900 μl/well of culture medium). After 2 h of incubation, the HCT-8 cell cultures were washed with PBS buffer and maintained in fresh culture medium for an additional 24 h. The cells were then fixed and permeabilized and blocked for non-specific binding as described above. Afterward, they were stained with Cy3-labeled Sporo-Glo^TM^ antibodies (Waterborne). The intensity of *C. parvum* infection in HCT-8 cells was measured by immunofluorescence microscopy, after the slides were mounted with a No-Fade Mounting Medium (Booster). Fifty visual fields were randomly selected and photographed under 200×, and the total number of parasites in each field was quantified using ImageJ 1.4.3.67^1^. The neutralization efficiency of immune sera or antibodies at different dilutions was calculated by comparing the number of parasites between treated groups and controls in three replicate analyses. The data generated were compared using Student’s *t*-test. Differences were considered significant at *p* ≤ 0.05.

## Results

### Expression of Recombinant INS-5 Protein

**FIGURE 1:**
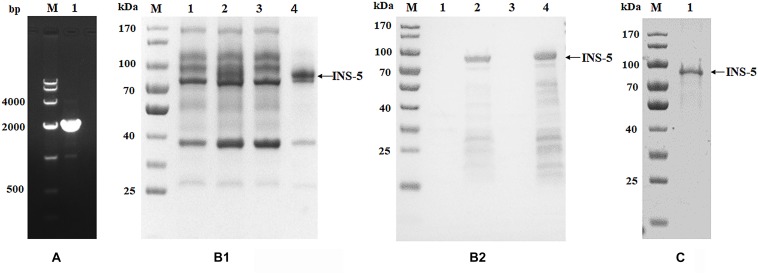
Cloning, expression, and purification of recombinant INS-5 of *Cryptosporidium parvum*. **(A)** The *cgd2_2760* gene was amplified by PCR from the genomic DNA of *C. parvum*. Lane M: DNA molecular marker; lane 1: *cgd2_2760* PCR product. **(B)** Expression of recombinant INS-5 in *E. coli* BL21 (DE3). B1 is the result of SDS-PAGE while B2 is that of western blot. Lane M: protein molecular weight marker; lane 1: bacterial lysate without isopropyl β-D-thiogalactoside (IPTG) induction; lane 2: bacterial lysate cultured for 5 h after IPTG induction; lane 3: supernatant of culture in lane 2; lane 4: pellet of culture in lane 2. **(C)** purification of recombinant INS-5 with a Ni-NAT affinity column. Lane M: protein molecular weight marker; lane 1: purified INS-5 observed by SDS-PAGE.

The *cgd2_2760* gene was successfully amplified by PCR from the genomic DNA of *C. parvum* using the forward and reverse primers with the added poly-histidine sequences (Figure 1A). The PCR product generated was cloned into a pET28a plasmid. The recombinant plasmid *cgd2_2760*-pET28a was transformed into *E. coli* BL21 (DE3) cells for the expression of INS-5 with a His-tag at both termini.

In SDS-PAGE analysis, the size of the INS-5 protein in the inclusion bodies from IPTG-induced *E. coli* BL21(DE3) cells transformed with the recombinant *cgd2_2760*-pET28a was in agreement with the predicted size of ∼78 kDa (Figure 1B1). Some lower-molecular-weight bands were observed in western blot analysis, probably due to early termination of the protein translation as a result of several rare *E. coli* codons (Figure 1B2). These proteins were identified as INS-5 fragments using MALDI-TOF-MS (data not shown). No expression of the recombinant INS-5 was detected in the supernatant of the *E. coli* lysate (Figure 1B). The best INS-5 expression as revealed by western blot analysis of lysates was achieved by culturing *E. coli* at 25°C for 5 h after induction with 0.5 mM IPTG. The recombinant INS-5 protein was purified using Ni-NTA affinity chromatography (Figure 1C).

### Immunoreactivity of Anti-INS-5 Antibodies

**FIGURE 2:**
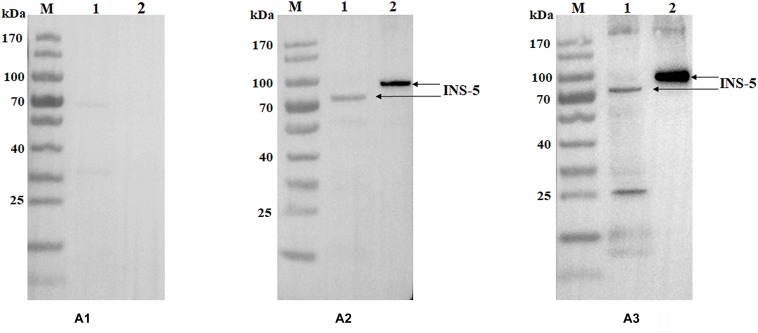
Western blot analyses of purified INS-5 and native INS-5 of *C. parvum*. **(A)** Polyclonal post-immune sera **(A2)** and antibodies **(A3)** were used to react with the recombinant INS-5 and the natural INS-5 in sporozoite extracts, with the pre-immune sera **(A1)** as the control. Lane M: protein molecular weight marker; lane 1: nature INS-5 extracted from sporozoites; lane 2: recombinant INS-5.

Polyclonal antisera and antibodies from the immunizations of rabbits with purified recombinant INS-5 protein were used in western blot analysis of the recombinant and native INS-5 proteins in sporozoite extracts. The recombinant INS-5 protein was recognized by polyclonal antisera and antibodies from INS-5-immunized rabbits but not by pre-immune sera (Figure 2). A single band was seen in western blot analysis of sporozoite extracts with antisera (Figure 2), suggesting that the antisera against the recombinant INS-5 could recognize the native INS-5. One extra band of ∼25 kDa was seen in western blot analysis of the native protein with purified IgG. The molecular weight of the natural INS-5 protein was slightly smaller (∼70 vs. 78 kDa) than that of the recombinant INS-5 protein, possibly because the native INS-5 protein was further processed in the parasites after its translation.

### Location and Expression of INS-5 Protein in *C. parvum*

**FIGURE 3:**
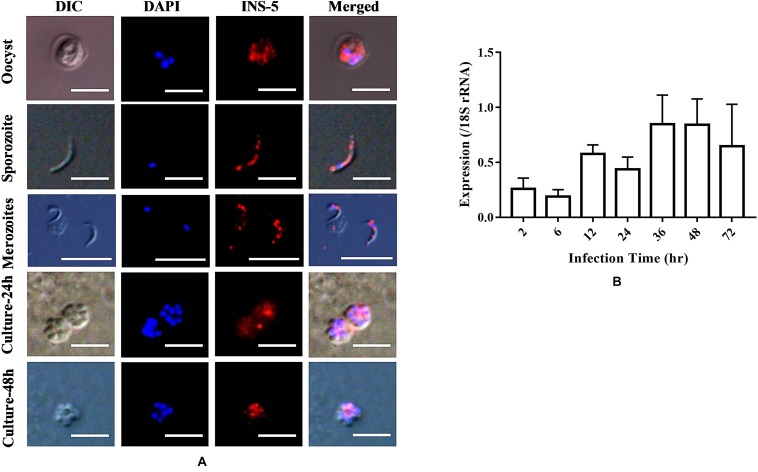
Location and expression level of the native INS-5 in *C. parvum*. **(A)** Location of INS-5 in oocysts, sporozoites, merozoites, and HCT-8 cells infected with *C. parvum* for 24 and 48 h. The images were taken under differential interference contrast (DIC). The red color shows the location of the INS-5 protein labeled by Alexa 594, and the blue color shows the position of the nuclei stained by DAPI. Scale bars = 5 μm. **(B)** Relative expression level of the *cgd2_2760* gene at different time points during *C. parvum* invasion as measured by quantitative PCR. The data on the expression of *C. parvum* 18S rRNA gene were used in data normalization. The data represent the mean ± SD calculated using three RNA extracts, each of which was analyzed in triplicate.

To assess the expression of the INS-5 protein in developmental stages of *C. parvum*, oocysts, sporozoites, merozoites, and *C. parvum*-infected HCT-8 cells cultured for 24 or 48 h were incubated with anti-INS-5 antibodies, labeled with fluorescence-conjugated secondary antibodies, and observed by immunofluorescent microscopy, using DAPI as the counterstain of cell nuclei (Figure 3A). As shown in Figure 3A, antibodies against INS-5 recognized sporozoites within oocysts (top panel). In sporozoites, the INS-5 protein was distributed in the entire sporozoite (Figure 3A, second panel). In HCT-8 cell culture, only part of the free merozoites or merozoites within meronts appeared to react with INS-5 antibodies, with the highest reactivity around the nuclei (Figure 3A, third, fourth, and bottom panels).

The mRNA transcriptional level of the *cgd2_2760* gene was evaluated using qPCR analysis of *C. parvum*-infected HCT-8 cells. The expression level of INS-5 was low at 2 and 6 h, increased at 12 h, and reached the highest at 36 and 48 h (Figure 3B).

### Neutralization Efficiency of INS-5 Antiserum Against *C. parvum* Invasion

**FIGURE 4:**
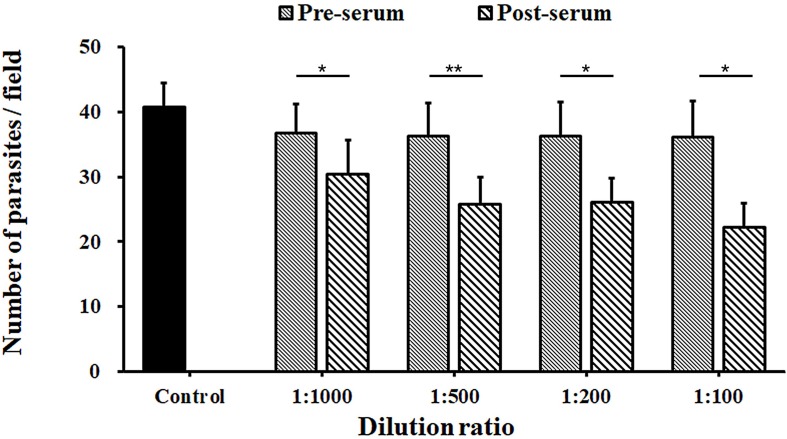
Neutralization efficiency of anti-INS-5 antiserum against *C. parvum* invasion. Oocysts of *C. parvum* were added to HCT-8 cell cultures in culture medium containing different dilutions (1:100, 1:200, 1:500, and 1:1,000) of pre-immune serum and post-immune serum, with medium alone as control. The number of developmental stages of the parasites was compared between antibody-treated groups and their corresponding controls. Data presented are mean ± SD from three replicate assays. The statistical significance of differences between treatment groups is indicated above the bars (*p < 0.05; **p < 0.01).

The inhibition rates of *C. parvum* invasion of HCT-8 cells by the INS-5 antiserum were 17.2% [36.7 ± 4.4 and 30.4 ± 5.3 per 200 × field for pre-immune and immune sera, respectively; *t*_(__2__)_ = 9.655, *p* = 0.011] at the dilution of 1:1,000, 29.0% [36.3 ± 5.1 and 25.8 ± 4.2 per 200 × field for pre-immune and immune sera, respectively; *t*_(__2__)_ = 11.434, *p* = 0.008] at the dilution of 1:500, 28.4% [36.3 ± 5.2 and 26.2 ± 3.7 per 200 × field for pre-immune and immune sera, respectively; *t*_(__2__)_ = 10.050, *p* = 0.010] at the dilution of 1:200, and 38.5% [36.1 ± 5.6 and 22.2 ± 3.7 per 200 × field for pre-immune and immune sera, respectively; *t*_(__2__)_ = 8.415, *p* = 0.014] at the dilution of 1:100 (Figure 4).

## Discussion

In this study, we have conducted one of the first studies of INS proteins in *Cryptosporidium* spp. Previously, only two INS proteins of *Cryptosporidium* spp., INS-15 and INS-20-15, had been characterized biologically (Xu et al., 2019; Zhang et al., 2019). We selected INS-5 in this study because it has homologs in most other *Cryptosporidium* species such as *Cryptosporidium hominis*, *Cryptosporidium muris*, and *Cryptosporidium ubiquitum* (Liu et al., 2016), indicating that it has conserved functions among *Cryptosporidium* spp.

Data from this study suggest that INS-5 plays some roles in the invasion and growth process of *C. parvum*. First, polyclonal antisera against INS-5 reduced the invasion of HCT-8 cells by *C. parvum* sporozoites by ∼38%. Second, the INS-5 had the highest expression in later time points of the *C. parvum* infection, the intracellular multiplication stages of the parasite, in agreement with qRT-PCR data in CryptoDB and recent RNA-seq data (Tandel et al., 2019). Third, the distribution of INS-5 is throughout the cytoplasm of the sporozoite.

In the neutralization assay, only a very modest inhibition rate was achieved by the anti-INS-5 antibodies. The subcellular localization of INS-5 could affect the neutralization efficiency of the antibodies, as if the protein has an intracellular location, the neutralization assay might not be the optimal way to determine its involvement during invasion. At the same time, intracellular apicomplexans are known to use multiple strategies to invade cells (Sibley, 2004) and the invasion of zoites inevitably requires the synergy of various molecules including surface antigens and apical proteins (Carruthers and Blackman, 2010). As a result, the *in vitro* neutralization ability of antibodies against individual invasion-related proteins could be limited (Fei et al., 2018). Further studies are needed to verify the neutralization capacity of polyclonal antibodies against INS-5.

The INS-5 protein appears to have post-translational processing. Lower-molecular-weight species were observed in SDS-PAGE and western blot analyses of the protein when INS-5 was expressed exogenously in *E. coli* BL21 (DE3). Some of the small INS-5 products could be due to early termination of the protein translation because of the existence of several rare *E. coli* codons in the recombinant *cgd2_2760*-pET28a plasmid. The use of the Rosetta (DE3) strain, however, failed to increase INS-5 expression in *E. coli*. Another reason for the existence of small INS-5 fragments could be due to post-translational proteolysis of INS-5. Toxolysin 4, an INS in *T. gondii*, was shown to undergo proteolytic maturation (Laliberté and Carruthers, 2011). In addition to the predicted native protein of ∼78 kDa, a band with a molecular mass of ∼25 kDa was seen in western blot analysis of sporozoite extracts with anti-INS-5 antibodies, which might be a processed product. Although this small fragment has not been observed in western blot analysis with antiserum, it is possible that the antibodies are directed at epitopes present on the parent molecule. Whether *Cryptosporidium* spp. further process INS *in vivo* requires validation. Multiple bands were also seen in the expression of human INS in *E. coli* (Chesneau and Rosner, 2000).

**FIGURE 5:**
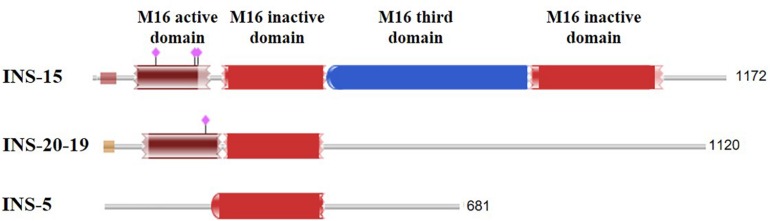
Comparison of the domain structure of several insulinase-like proteins of *C. parvum* that have been studied thus far. The purple diamonds indicate the predicted active site of the M16 peptidase.

It appears INS-5 may not have any classic INS activities. Although it is a member of the INS family, INS-5 contains only one inactive M16 domain, compared with four domains in classic INS, including the first domain that contains the enzyme activity site (Figure 5). Previous studies showed that over half of the *Cryptosporidium* INS members lack the active Zn-chelating “HXXEH” motif and thus are likely to be catalytically inactive copies (Abrahamsen et al., 2004). These INS, including INS-5, may participate in protein–protein interactions during life cycle, but their precise role in *Cryptosporidium* life cycle needs further research. INS genes in *C. parvum* are expressed at various levels and with different patterns (Mauzy et al., 2012), implying that these INS proteins play different biological functions at various life cycle stages. This has been supported by recent studies of INS-15 and INS-20-19, which have different expression patterns (Xu et al., 2019; Zhang et al., 2019). Another INS that has four domains of classic INS, INS-4 encoded by the *cgd2_930* gene, has been identified as a putative rhoptry protein of sporozoites (Sanderson et al., 2008). In the life cycle of *C. parvum*, the biological functions of these INS proteins could be different from INS-5.

In summary, this preliminary study represents our initial attempts in characterizing the functions of INS-5 in *C. parvum*. Although INS-5 was observed to be expressed in the invasive stages of *C. parvum* by immunofluorescent microscopy and its gene showed the highest expression in later time points of the *C. parvum* infection by qRT-PCR analysis, the specific location of the protein remains unclear. Ultrastructural analysis is needed to determine the subcellular location of INS-5. Gene ablation or silencing is also needed in further assessing the role of INS-5 in the invasion and growth of *C. parvum*. This would allow better understanding of the developmental regulation and biological functions of INS proteins in *Cryptosporidium* life cycle stages.

## Data Availability Statement

All datasets generated for this study are included in the article/supplementary material.

## Author Contributions

YF and LX conceived and designed the experiments. NN performed the experiments. RJ, YG, NL, and HW provided technical assistance. NN, YF, and LX analyzed the data. NN, RJ, YF, and LX wrote the manuscript.

## Conflict of Interest

The authors declare that the research was conducted in the absence of any commercial or financial relationships that could be construed as a potential conflict of interest.

## Footnotes

1 https://imagej.nih.gov/ij/
